# The Challenges of Inflation in Europe and the US

**DOI:** 10.1007/s10272-022-1031-z

**Published:** 2022-04-08

**Authors:** Forum Introduction

## Abstract

Inflationary pressures and uncertainties related to the severity and duration of the coronavirus pandemic have been growing steadily in the last year. Different approaches to supporting labour force participation in Europe and the United States have led to different inflationary outcomes. In addition, the Russian war against Ukraine, which began on 24 February, has provided a definitive answer to questions about whether rising inflation is just temporary. The sharp increase in already volatile food and energy prices is presenting monetary policymakers with new challenges. The authors in this Forum consider how to respond to rising inflation, discuss why inflation is so hard to predict and examine whether long-term inflation expectations have de-anchored.

